# Ionic transport of proton-conducting ammonium vanadate salt in blends of polyvinyl alcohol and sodium alginate for electrochemical applications

**DOI:** 10.1038/s41598-025-31055-5

**Published:** 2025-12-05

**Authors:** M. Elakkiya, R. Jansi, M. S. Revathy, J. Kanimozhi, Saravanan Rajendran, Manikandan Ayyar, Prabhu Paramasivam, Mohamed Yusuf

**Affiliations:** 1 Department of Chemical Engineering, School of Bio, Chemical and Processing Engineering, Kalasalingam Academy of Research and Education, Krishnankoil, Tamil Nadu 626126 India; 2 Department of Science and Humanities, AAA College of Engineering and Technology, Amathur, Sivakasi, Tamil Nadu 626 005 India; 3 Department of Physics, School of Advanced Sciences, Kalasalingam Academy of Research and Education, Krishnankoil, Virudhunagar, Tamil Nadu 626126 India; 4 Department of Biotechnology, School of Biochemical and Processing Engineering, Kalasalingam Academy of Research and Education, Krishnankoil, Tamil Nadu 626126 India; 5 Instituto de Alta Investigación, Universidad de Tarapacá, 1000000 Arica, Chile; 6 Department of Chemistry, Karpagam Academy of Higher Education, Coimbatore, Tamil Nadu 641021 India; 7 Centre for Material Chemistry, Karpagam Academy of Higher Education, Coimbatore, Tamil Nadu 641021 India; 8 Centre for Research Impact & Outcome, Chitkara University Institute of Engineering and Technology, Chitkara University, Rajpura, Punjab 140401 India; 9 Department of Peace and Development Studies, Njala University, Bo Campus-18, Bo , Sierra Leone

**Keywords:** Flexible polymer electrolytes, Sodium alginate, PVA, Ionic conductivity, NH₄VO₃, XRD, FTIR, Chemistry, Energy science and technology, Materials science

## Abstract

Exploring highly foldable batteries with no safety hazard is a vital task for the realization of portable, wearable, and implantable electric devices. Owing to these concerns, developing solid-state batteries is one of the most promising routes to achieve this aspiration. Because of the excellent flexibility and process ability, Sodium alginate blends polyvinyl alcohol-based electrolytes possess great potential to pack high energy density flexible batteries, however, suffers the various intrinsic shortcomings such as inferior ionic conductivity, a high degree of crystallinity, and lack of reactive groups. In this present work, polymer electrolyte films based on NaAlg blend PVA doped with NH_4_VO_3_ salt were prepared by solution casting method. X-ray diffraction (XRD) explains that the enhancement of conductivity is affected by the degree of crystallinity. Fourier transform infrared (FTIR) spectroscopy analysis confirms the interaction between polymers and salt. For NaAlg/PVA system, a sample containing 15 wt% of NH_4_VO_3_ possesses the highest ionic conductivity of 0.67 × 10^− 5^ S cm^− 1^. Several electrical and electrochemical characteristics of the prepared electrolytes were examined, including impedance, dielectric behavior, transference number, electrochemical stability window, energy density, specific capacitance (Cs), and power density. The ionic conductivity of the synthesized solid biopolymer electrolyte (SBE) system was found to be influenced by ion mobility (µ) and the diffusion coefficient (D). Hence, the aforementioned results indicate that the developed SBE system holds strong potential for application in electrochemical energy storage and conversion devices such as proton batteries, supercapacitors, and fuel cells.

## Introduction

Polymer electrolytes are considered as viable materials for emerging energy storage and conversion technologies, including solid-state batteries, supercapacitors, and fuel cells. Their favourable characteristics-such as mechanical flexibility, thermal stability, and design adaptability—make them promising alternatives to conventional liquid electrolytes, which suffer from issues like leakage, volatility, and chemical instability^1,2^. Among these, solid polymer electrolytes (SPEs) offer significant advantages by enabling safer, leak-proof, and more compact devices^3^.

The development of polymer blend systems combining synthetic and natural polymers has gained momentum in recent years due to their enhanced ion transport properties, environmental compatibility, and film-forming ability. Poly(vinyl alcohol) (PVA) is a widely studied synthetic polymer known for its semi-crystalline structure, hydrophilicity, and excellent dielectric strength. Its hydroxyl-rich backbone allows complexation with salt ions, promoting ionic mobility^4^. Blending PVA with sodium alginate (NaAlg)—a biopolymer derived from brown seaweed containing carboxyl functional groups—further enhances its electrochemical and mechanical performance. The resulting polymer matrix supports improved ion hopping through both hydroxyl and carboxyl coordination sites, making it suitable for flexible and biodegradable electrochemical systems^5,6^.

In recent years, research efforts in polymer electrolytes have predominantly centred on lithium-ion (Li⁺) and sodium-ion (Na⁺) battery systems because of their promising energy density and established technological base^7^. These studies have explored polymer hosts and biopolymer blends designed to support Li⁺/Na⁺ transport^8–10^. Nevertheless, these systems face persistent limitations—such as narrow electrochemical stability windows, high interfacial resistance with electrodes, mechanical fragility under deformation (for wearable devices), and sustainability issues linked to lithium or sodium resources^11–13^.

To improve ionic conductivity, such polymer blends are typically doped with ammonium salts, which act as sources of NH₄⁺ cations, capable of moving through the polymer network via segmental motion and hydrogen bonding interactions^14^. Ammonium-based salts like NH₄Cl, NH₄NO₃, and NH₄SCN have been widely used in various polymer matrices, although their conductivities are often limited by ion pairing or inadequate salt dissociation—leading to values lower than 10 –6 S/cm^15–17^. The challenge remains to identify suitable ammonium salts that can promote both high ionic dissociation and polymer compatibility.

In this context, ammonium metavanadate (NH₄VO₃) presents a unique advantage (i.e.) besides NH₄⁺ ions, VO₃⁻ anions, which are redox-active and may contribute to additional charge transport or even pseudocapacitive behavior. The dual ionic nature and redox properties of NH₄VO₃ can potentially enhance ionic conductivity and structural robustness^18,19^. Moreover, vanadium-based dopants have been shown to improve the mechanical integrity, thermal resistance, and electrochemical functionality of polymer systems^20^.

Several methods exist for the fabrication of polymer electrolytes, including hot pressing, sol–gel processing, electrospinning, and solution casting. Among these, the solution casting technique is particularly attractive due to its low cost, ease of fabrication, and ability to produce homogenous, flexible thin films with well-dispersed salt content and controlled morphology^21^. It is especially effective for multi-component systems where polymer-salt interactions must be carefully tuned.

Polyvinyl alcohol (PVA) blended with sodium alginate (NaAlg) has been widely studied as a polymer host for solid electrolytes, and several reports confirm that the conductivity often remains below 10⁻⁶ S cm⁻¹ for many salt concentrations. Cyriac et al. demonstrated that introducing sodium iodide (NaI) into PVA–NaAlg increased conductivity, but values at lower salt loadings were confined to the 10⁻⁷–10⁻⁶ S cm⁻¹ range, approaching ~ 10⁻⁶ S cm⁻¹ only at optimum composition^22,23^. Jansi et al. investigated ammonium nitrate (NH₄NO₃) addition and found that a maximum of ~ 1.05 × 10⁻⁶ S cm⁻¹ occurred for the 60:40:15 blend ratio, while reduced salt fractions consistently gave conductivities below 10⁻⁶ S cm⁻¹^24^. In another study, researchers working with ammonium chloride (NH₄Cl) observed a peak of ~ 1.01 × 10⁻⁶ S cm⁻¹ at 15 wt%, with all lower concentrations remaining in the sub-10⁻⁶ regime^25^. Sheela et al. reported that doping with potassium chloride (KCl) yielded conductivity values between 10⁻⁸ and 10⁻⁶ S cm⁻¹ at ambient temperature, with the majority of compositions never surpassing the 10⁻⁶ mark^26^. Likewise, studies on ammonium thiocyanate (NH₄SCN) incorporated into PVA–NaAlg showed that initial salt additions gave conductivities in the 10⁻⁷–10⁻⁶ S cm⁻¹ range, and only higher concentrations pushed the values beyond 10⁻⁶ S cm⁻¹^27^.

As a result, there is growing interest in alternative ionic conduction systems, particularly those based on protonic or ammonium (NH₄⁺) charge carriers. These species offer potential advantages including lighter weight, greater abundance, and favourable hydrogen-bond networks that facilitate ion hopping^28^. Ammonium-doped polymer electrolytes in particular promise flexible, environmentally benign architectures. However, many of these ammonium-ion systems still suffer from relatively low ionic conductivities (often < 10⁻⁶ S cm⁻¹) and limited electrochemical functionality^29^.

The proton conduction mechanism in ammonium salt–based systems arises from the weakly bonded proton (H⁺) in the NH₄⁺ ion, which facilitates ionic motion through the proton-hopping mechanism. Abdullah et al. (2018) demonstrated that incorporation of NH₄NO₃ into a methylcellulose–PVA blend produced a proton-conducting polymer electrolyte, where one of the four protons attached to the nitrogen atom is loosely bound and capable of hopping between neighboring electronegative sites^30^. Similarly, Hemalatha et al. (2019) reported that ammonium salts act as effective proton donors, as the loosely bound hydrogen in NH₄⁺ migrates through hydrogen-bonded pathways in the polymer matrix, enhancing ionic conductivity^31^.

The ionic conductivity value obtained in this study aligns with previously reported proton-conducting biopolymer electrolytes. In the report of MC–PVA: NH₄NO₃ 20 wt% achieved a conductivity of 7.39 × 10⁻⁵ S cm⁻¹^30^, while Nizam et al. (2025) reported an ac conductivity of 1.01 × 10⁻⁵ S cm⁻¹ for Alginate–PVA: NH₄I bio-polymer electrolytes prepared by solution casting^32^.

In view of aforementioned challenges, the present work introduces a novel solid polymer electrolyte system composed of NaAlg–PVA blended matrix doped with ammonium metavanadate (NH₄VO₃). This material strategy offers several advantages: (i) the NH₄⁺ ion acts as a mobile charge carrier enabling protonic or ammonium ion conduction; (ii) the VO₃⁻ moiety brings redox-active centres and structural reinforcement, thereby affording the possibility of pseudocapacitive behaviour or improved dielectric response; (iii) the biopolymer matrix grants flexibility, biodegradability and potentially lower cost compared to conventional Li⁺/Na⁺ systems. Thus, the reported NaAlg–PVA–NH₄VO₃ composite electrolyte presents a sustainable alternative to Li⁺/Na⁺ oriented systems, offering enhanced ionic mobility, mechanical robustness, and multifunctional electrochemical behaviour.

Herein we report the influence of varying concentrations of ammonium metavanadate (NH₄VO₃) on the ionic conductivity, structural properties, dielectric behavior and electrochemical performance of PVA–NaAlg solid blend polymer electrolytes.

## Material and method

**Fig. 1:**
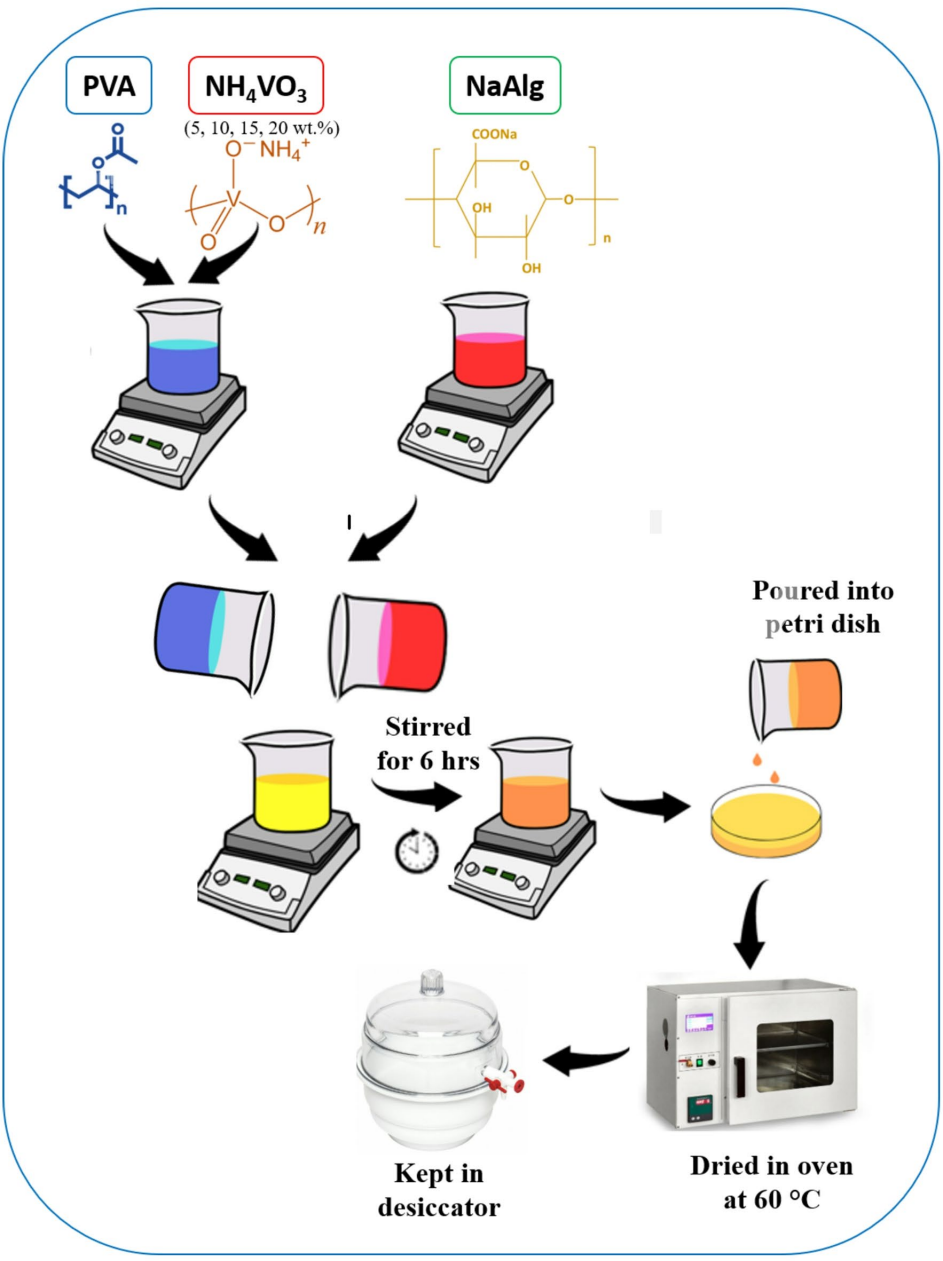
Schematic illustration of the preparation of the PNV polymer blend electrolytes.

Polyvinyl alcohol (PVA, MW = 125000 g/mol), sodium alginate (NaAlg, MW = 194.18 g/mol), and ammonium metavanadate (NH₄VO₃, MW = 116.98 g/mol) were obtained from Otta Chem Pvt Ltd, Sigma-Aldrich, and Avra Chem Ltd, respectively. Distilled water served as the solvent. The optimized PVA: NaAlg blend ratio reported in our earlier study^17^ was adopted for preparing polymer electrolytes incorporating different concentrations of NH₄VO₃ (5, 10, 15, and 20 wt%). The PVA/NH₄VO₃ solution was first prepared in distilled water, while NaAlg was dissolved separately. Both solutions were then combined and stirred continuously for about 6 h to ensure uniform mixing. The homogeneous solution was cast into petri dishes and dried in an oven at 60 °C to obtain thin membranes. The prepared films were subsequently stored in a desiccator. The designations of the samples with varying NH₄VO₃ loadings are summarized in Table 1. The resulting membranes exhibited thicknesses in the range of 0.006–0.015 mm, as measured using a screw gauge. A schematic representation of the synthesis procedure for PVA/NaAlg–NH₄VO₃ polymer electrolytes is shown in Fig. 1.

**Table 1:** Sample code assignment for prepared polymer electrolytes.

Sample	Code
PVA: NaAlg	PN
PVA: NaAlg: 5 wt% NH_4_VO₃	PNV 1
PVA: NaAlg: 10 wt% NH_4_VO₃	PNV 2
PVA: NaAlg: 15 wt% NH_4_VO₃	PNV 3
PVA: NaAlg: 20 wt% NH_4_VO₃	PNV 4

### Characterization techniques

The structural characteristics of the amorphous and crystalline phases of the solid polymer electrolyte (SPE) were examined using a Bruker X-ray diffractometer (λ = 1.54 Å) with a scanning rate of 5° per minute over the 2θ range of 10° to 60°. Fourier Transform Infrared (FTIR) spectroscopy, performed with the SHIMADZU IR Tracer 100, was employed to monitor the incorporation of NH₄VO_3_ into the polymer blend. The spectra were recorded in the wavenumber range of 4000–500 cm⁻¹ to analyze the interactions among PVA, NaAlg, and NH₄VO_3_. Ionic conductivity measurements were conducted using a HIOKI 3532-50 LCR Hi-Tester within the frequency range of 42 Hz to 1 MHz. For electrochemical analysis, cyclic voltammetry (CV) was carried out using a CH-Instrument Model 6008e.

### Fabrication of electrode

The first stage in preparing the EDLC is the development of carbon electrodes. N-methyl pyrrolidone (NMP), poly(vinylidene fluoride) (PVdF), and activated carbon (obtained from SANWA Components, Inc.) were utilized in an 8:1:1 weight ratio. Using a mortar and pestle, PVdF and activated carbon were thoroughly combined with NMP to create a homogenous slurry. After evenly coating nickel foil with this slurry, it was dried for 12 h at 80 °C. In order to eliminate any remaining moisture, the resultant electrodes were then kept in a desiccator filled with silica gel.

## Result and discussion

### XRD analysis

**Fig. 2:**
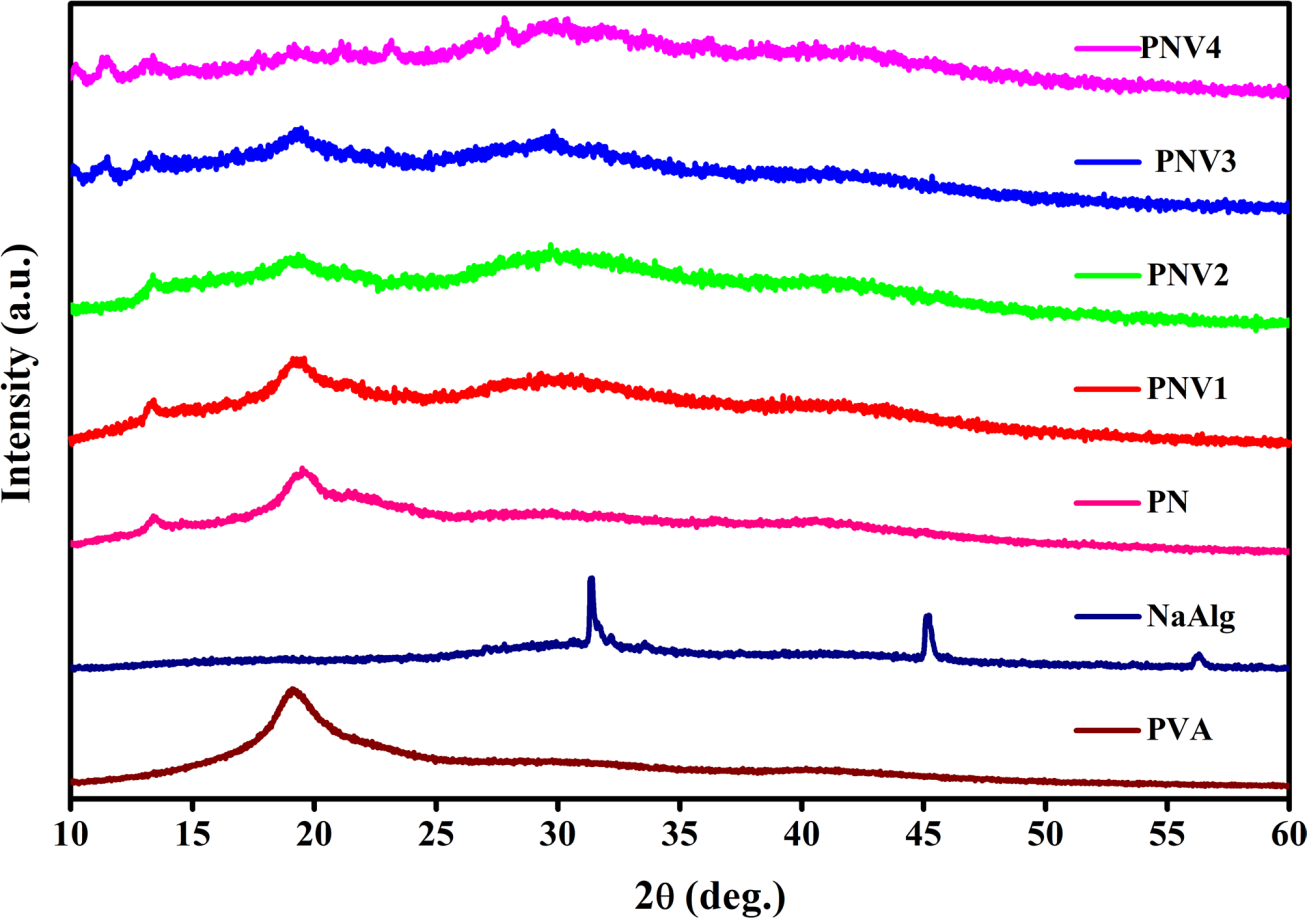
X-ray diffractograms of pure PVA, Pure NaAlg, PVA-NaAlg(PN) blend, and PNV (PVA: NaAlg: NH_4_VO_3_(5, 10, 15, 20 wt%) polymer blend electrolytes.

The XRD pattern of Pure PVA, NaAlg and blended electrolytes (PNVI-PNV4) were shown in Fig. 2. It exhibits a broad diffraction peak (indicative of its semicrystalline nature) at 2θ ≈ 19.5–20° for pure PVA and intense peaks of pure NaAlg at 13°, 31.42°, and 45.14° are attributed to polysaccharide structure. Upon blending to make the PN sample, the smaller peak intensities and widener profiles denote the breakdown of crystalline areas, and are explained by stretching the initial order of the matrix by interpolymer interactions^33^. Upon doping with NH₄VO₃, the relative intensity of the peaks decreases due to the distortion of the polymer structure and enhancing the amorphous nature. It can be observed that the intensity of peak decrease from PNV1 to PNV3 due to strong intermolecular interactions. Notably, PNV3 exhibits the lowest peak intensity, suggesting the highest degree of amorphization, which is favorable for ion mobility. On further addition of the salt, the intensity of peaks increases for PNV4, which indicates the increase in crystallinity. The rise in peak intensity suggests that at this higher salt level, the polymer host reaches its saturation limit, causing a portion of the NH₄VO₃ to segregate into ordered regions. This leads to the return of crystalline features due to localized salt accumulation or slight structural ordering within the polymer chains^34–36^. The presented structural change between the crystalline and the amorphous states is quite consistent with previous experimental and simulation studies of solid polymer electrolytes and has demonstrated that highly amorphous systems such as PNV3 have better ionic conductivity^37^.

Equation (1) is employed for calculating the percentage crystallinity ($\:{\chi\:}_{c}$) of the electrolytes.

1 $$\:{\chi\:}_{c}=\frac{{A}_{c}}{{A}_{c}+{A}_{a}}\times\:100\: (\%)$$

where A_c_ → area under the crystalline peaks, A_a_ → area under the amorphous peak. The values are represented in Table 2.

**Table 2:** Degree of crystallinity calculated from XRD data.

S.No.	Composition	Crystallinity (%)	Amorphous (%)
1.	PVA	41.68	58.32
2.	NaAlg	19.6	80.4
3.	PN	22	78
4.	PNV1	17.9	82.1
5.	PNV2	19.1	80.9
6.	PNV3	14.8	85.8
7.	PNV4	20.5	79.5

### FTIR analysis

**Fig. 3:**
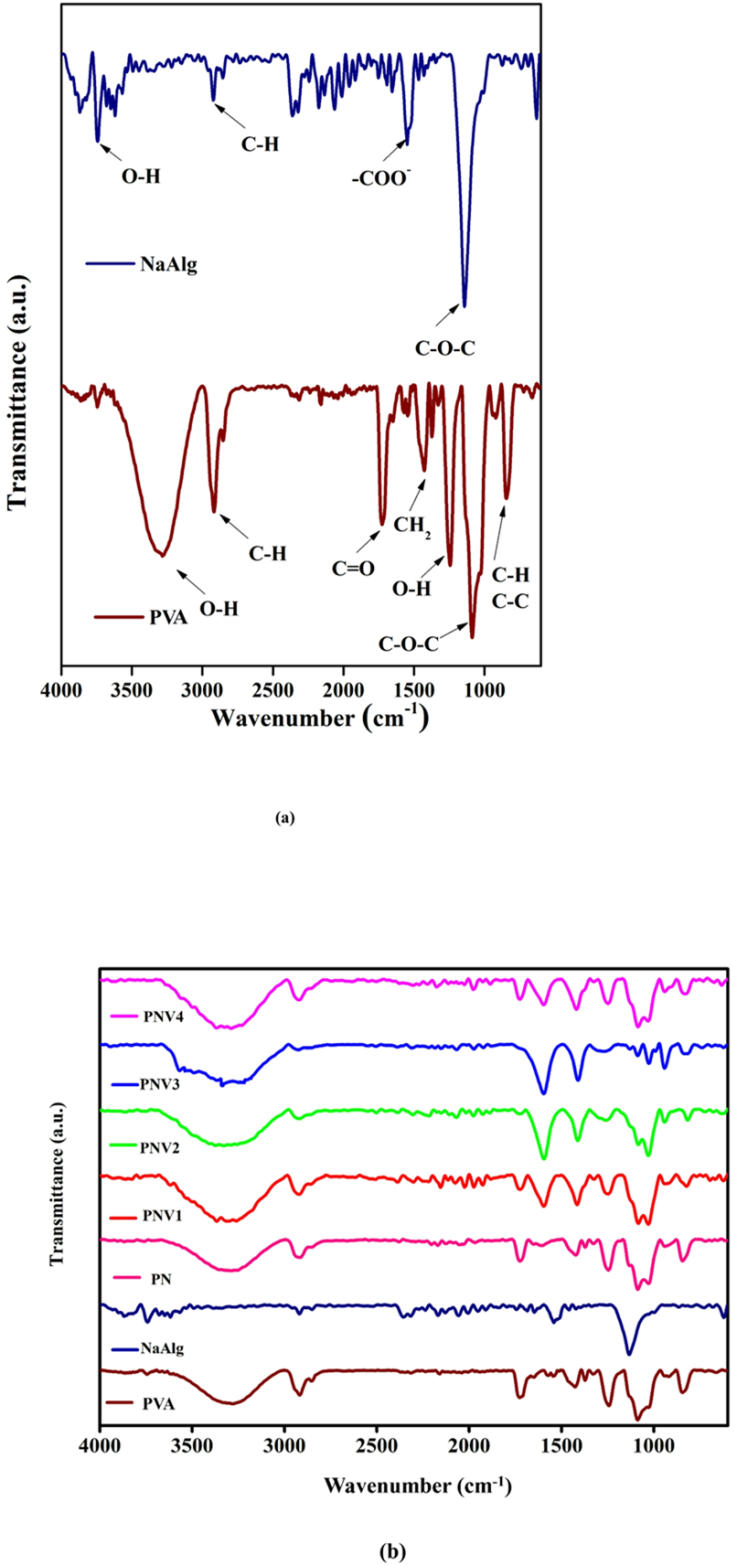
(**a**). FTIR spectra of pure PVA and Pure NaAlg, (**b**) pure NaAlg, PVA-NaAlg(PN) blend, and PNV (PVA: NaAlg: NH_4_VO_3_ (5, 10, 15, 20 wt%) polymer blend electrolytes.

FTIR spectra of pure PVA, pure NaAlg, and PVA: NaAlg: NH_4_VO_3_ (5, 10, 15, 20 wt%) membranes are illustrated as PN, PNV systems in Fig. 3. These spectra demonstrates the existence and interconnection of major functional groups and are presented in Table 3. A wide and high band seen approaching 3309 cm^− 1^ in all the samples was found to be due to OH- stretching vibration of both PVA and NaAlg and was ascribed to hydrogen bonding between Hydroxyl groups of PVA and NaAlg. The doped samples (PNV series) shifted this band, which reflects an increased hydrogen bonding and polymer-salt interaction^38,39^.

The stretching vibration of C-H in the range of ~ 2916–2917 cm^− 1^ is the indication of the presence of aliphatic chains of PVA, which do not change during blending or doping. This is because the C = O stretching vibration at around 1720–1725 cm^− 1^ due to the uronic acid units in sodium alginate did not disappear, which is evidence of retention of backbone intactness across the samples^14^. A major change is the change of the COO- asymmetric stretching of vibration at 1540 cm^− 1^ in pure NaAlg to 1594 cm^− 1^ in the doped samples, which indicates complexation of the carboxylate group of Alginate with ammonium ions of NH_4_VO_3_^12,40^.

**Table 3:** Vibrational assignments of PN) blend, and PNV series - PVA: naalg: NH_4_VO_3_ (5, 10, 15, 20 wt%) polymer blend electrolytes.

Vibrational mode	Band assignments			
	NaAlg (cm⁻¹)	PVA (cm⁻¹)	NaAlg: PVA (PN) (cm⁻¹)	NaAlg: PVA: NH₄VO₃ (PNV series) (cm⁻¹)
O–H stretching	~ 3280	~ 3286	~ 3309	~ 3309
C–H stretching	–	~ 2917	~ 2916	~ 2916
C = O stretching	~ 1720	–	~ 1725	~ 1725
COO⁻ asymmetric stretching	~ 1540	–	–	~ 1594
CH₂ bending	~ 1421	~ 1418	~ 1418	~ 1418
C–O–C stretching	~ 1125	1249,1091,1029	1252, 1086, 1025	1252, 1086, 1025
C–C bending	–	~ 929	~ 929	~ 929
C–H bending	–	~ 838	~ 838	~ 838

Also, the bending vibrations of CH_2_ at ~ 1418–1421 cm^− 1^ and the C-O-C stretching vibrations observed in the region of 1252 –1025 cm^− 1^ indicate the structure of the PVA-NaAlg matrix. These are peaks that do not experience dramatic change in position but they experience slight broadening in the doped films in agreement with the polymer-salt interactions that enhance amorphicity without disrupting the polymer chains^39,40^. Backbone vibration of base polymers is also confirmed by the C-C bending mode (929 cm^− 1^) and C-H bending modes (838 cm^− 1^)^38^. These observed spectral shifts, as well as the broadening, are revealing the effective mixing and complexation between NaAlg, PVA, and NH_4_VO_3_ and thereby achieving better values in amorphous content and enhancing the behavior of ionic transportation as observed in earlier works of PVA-based electrolyte systems in the doped forms of ammonium salts, and vanadates^12,41^.

### AC impedance spectroscopy

For the determination of impedance, i.e. Z∗(ω) = Z′ +jZ″ where Z′ and Z″ are the real and imaginary parts of the impedance, the polymeric films were sandwiched in between silver as blocking electrodes.

#### Nyquist plots

**Fig. 4:**
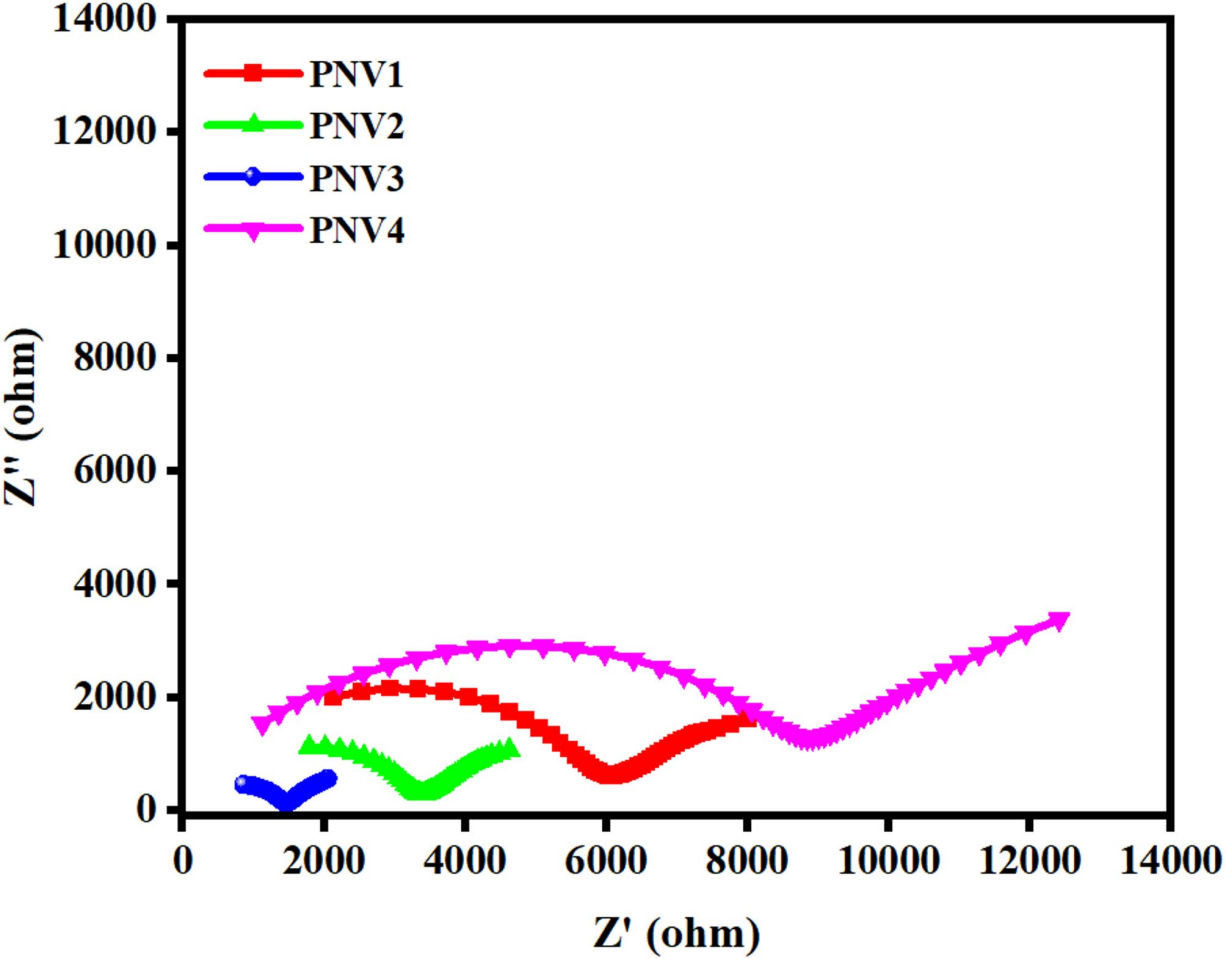
Nyquist curve for PVA: NaAlg: NH_4_VO_3_ (at room temperature).

The Nyquist plots of NaAlg: PVA: NH₄VO₃ (5 wt% to 20 wt%) polymer electrolyte films in Fig. 4 shows the two distinct depressed semicircles followed by a low-frequency tail which affirms to the coexistence of bulk and interfacial relaxation processes. the high-frequency arc pertains to the bulk ionic conduction and mid-frequency arc associate with inter-domain or electrode-electrolyte interfacial resistance^42,43^. The ionic conductivity of all the polymer electrolytes has been evaluated using the equation

2 $$\:\sigma\:=\frac{t}{{R}_{b}\:\text{A}}$$

where t is the thickness and A is the contact area of the electrolyte film. The Warburg-like tail (low frequency) line reflects behavior on capacitive blocks at electrodes and the semicircle is the bulk resistance of the electrolyte (R_b_). The PNV3 15 wt% NH₄VO₃ has the least diameter of the semicircle of the compositions analyzed, which implies that it has the lowest concentration of R_b_ and has ionic conductivity of 0.67 × 10^− 5^ S cm^− 1^^43^. This agrees with studies that demonstrate that conductivity proceeds to increase with salt concentration as far as it permits a lot and then excess salt makes partners remain with ions or cluster together that reduces free ion movement and increases resistance^44^. The presence of the spike at the low frequencies provides second evidence that the diffusion method is non-Faradaic and ionic dependent under blocking electrode principle^42^. Altogether, the impedance measurements are in favour of 15 wt% NH₄VO₃ as the ideal loading in providing flexibility of the polymerised chain, dissociation of the ions and charge transport to move freely in order to ensure high performance of electrolyte. The diffusion coefficient(D), mobility(µ) and number of charge carriers(N) were estimated and tabulated in the Table 4 using the following relation,

3 $$\:D=\:\frac{2\pi\:{f}_{max}{d}^{2}}{{32(\text{tan}{\delta\:}_{max})}^{3}}\:\:\:\:\:\:\:\:\:\:\:\:\:\:\:\:\:\:\:\:\:\:\:\:\:\:\:\:\:\:\:\:\:\:\:\:\:\:\:\:\:\:\:\:\:\:\:\:\:$$

4 $$\:\mu\:=\frac{{\sigma\:}_{dc}}{Nq}\:\:\:\:\:\:\:\:\:\:\:\:\:\:\:\:\:\:\:\:\:\:\:\:\:\:\:\:\:\:\:\:\:\:\:\:\:\:\:\:\:\:\:\:\:\:\:\:\:\:\:\:\:\:\:\:\:\:\:\:\:\:\:\:\:\:\:\:\:\:\:$$

5 $$\:N=\:\frac{{\sigma\:}_{dc}{k}_{B}T}{D{q}^{2}}\:\:\:\:\:\:\:\:\:\:\:\:\:\:\:\:\:\:\:\:\:\:\:\:\:\:\:\:\:\:\:\:\:\:\:\:\:\:\:\:\:\:\:\:\:\:\:\:\:\:\:\:\:\:\:\:\:\:\:\:\:\:$$

Where, *D* → diffusion coefficient of ions (cm²/s); *f*_*max*_ → frequency at which dielectric loss (tan δ) shows a maximum (Hz); *d* →thickness of the electrolyte film (cm); tan *δ*_*max*_ →maximum dielectric loss tangent; µ → Ionic mobility (cm²/V·s); *σ*_*dc*_→ dc ionic conductivity (S/cm); N → number of charge carriers per unit volume (cm⁻³); q → Charge of a single ion (1.6 × 10⁻¹⁹ C); *k*_*B*_ → Boltzmann constant (1.38 × 10⁻²³ J/K); *T* → absolute temperature (K).

**Table 4:** Electrical and transport parameters of naalg: PVA: NH₄VO₃ polymer electrolyte films.

Sample code	_dc_ (S/cm)	(s)	D (cm^− 2^s^− 1^)	(cm^−2^^−1^ s^−1^)	*N* (cm^− 3^)
PN	5.592 × 10^− 7^	5.4 × 10⁻⁵	5.4 × 10⁻⁵	5.4 × 10⁻⁵	2.5 × 10²⁰
PNV1	1.58 × 10^− 6^	1.89 × 10^− 5^	6.70×× 10^− 11^	2.56 × 10^− 9^	2.54 × 10^21^
PNV2	3.16 × 10^− 6^	1.01 × 10^− 5^	1.14 × 10^− 10^	4.37 × 10^− 9^	2.9 × 10^21^
**PNV3**	**0.67 × 10** ^**− 5**^	**6.76 × 10** ^**− 6**^	**2.14 × 10** ^**− 10**^	**8.17 × 10** ^**− 9**^	**3.23 × 10** ^**21**^
PNV4	7.76 × 10^− 7^	5.55 × 10^− 5^	2.17 × 10^− 11^	8.34 × 10^− 10^	3.54 × 10^21^

Significant values are in bold.

### Interpretation of CNLS fitting

The impedance spectra of the NaAlg–PVA: NH₄VO₃ polymer blend films in Fig. 4 were analyzed using complex nonlinear least-squares (CNLS) fitting using ZView software to evaluate the effect of salt concentration on ion transport behaviour. The spectra were fitted with the equivalent electrical circuit model (R_s_-(R_b_||CPE_b_)-(R_gb_||CPE_gb_)- CPE_el_), where R_s_ is the series/contact resistance (Ω), R_b_ is the bulk resistance of the conductive domains (Ω), and CPE_b_ is the constant-phase element (CPE) representing non-ideal bulk capacitance, R_gb_ is the grain-boundary / inter-domain / interfacial resistance (Ω), and CPE_gb_ is the corresponding CPE, and CPE_el_ represents the low-frequency, electrode-related non-ideal capacitance (blocking behaviour and any diffusion tail) which accounts for bulk and interfacial contributions to the total impedance. The parameters obtained from the fitting are summarized in Table 5. The fitted values clearly shows that the bulk resistance (R_b_) decreases as the NH₄VO₃ content increases from 5 wt% (PNV1) to 15 wt% (PNV3), indicating enhanced ionic motion within the polymer matrix. The 15 wt% film exhibits the smallest (R_b_) value (≈ 134 Ω) and the shortest relaxation time ((τ_b_ ≈ 4 × 10^− 6^) s), confirming that this composition supports the fastest ion migration and the most efficient segmental dynamics. Beyond this optimum level, at 20 wt% (PNV4), (R_b_) rises sharply and the overall arc broadens, which can be attributed to ion–ion interactions and the formation of aggregated clusters that restrict charge movement^45^.

The capacitance associated with the bulk process (C_b_) increases gradually with salt loading up to 15 wt%, reflecting greater charge storage capability due to improved ion dissociation and the increased availability of polar sites. However, the interfacial elements (R_gb_) and (C_gb_) become unstable at higher loading, consistent with the emergence of a secondary semicircle in the Nyquist plot, suggesting increased inter-domain polarization and ion accumulation at boundaries^46^. Thus, the CNLS analysis demonstrates that a moderate NH₄VO₃ concentration (15 wt%) provides the best compromise between ion dissociation and polymer-chain flexibility, resulting in low resistance and a faster dielectric relaxation process. Higher salt content leads to clustering and restricted mobility, while lower loading yields incomplete ion dissociation and reduced conductivity.

**Table 5:** CNLS fitting parameters for NaAlg–PVA: NH₄VO₃ electrolytes.

Sample	*R*_s_ (Ω)	*R*_b_ (Ω)	CPE_b_ (F)	*R*_gb_ (Ω)	CPE_gb_ (F)	τ_b_ (s)	τ_gb_ (s)
PNV1 (5 wt%)	5058.05	1498.56	6.53 × 10⁻⁹	–9.12 × 10⁴	12.37	1.0 × 10⁻⁵	–1.13 × 10⁶
PNV2 (10 wt%)	3088.17	665.10	1.96 × 10⁻⁸	–7.64 × 10⁴	46.18	1.3 × 10⁻⁵	–3.53 × 10⁶
PNV3 (15 wt%)	1343.58	133.99	3.35 × 10⁻⁸	853.52	–1.00 × 10⁻⁶	4.0 × 10⁻⁶	–1.27 × 10⁻³
PNV4 (20 wt%)	5325.04	3842.86	2.39 × 10⁻⁹	–1.47 × 10⁵	9.86	9.2 × 10⁻⁶	–1.44 × 10⁶

#### Conduction spectra

##### (a) AC conductivity and ion transport behavior

**Fig. 5:**
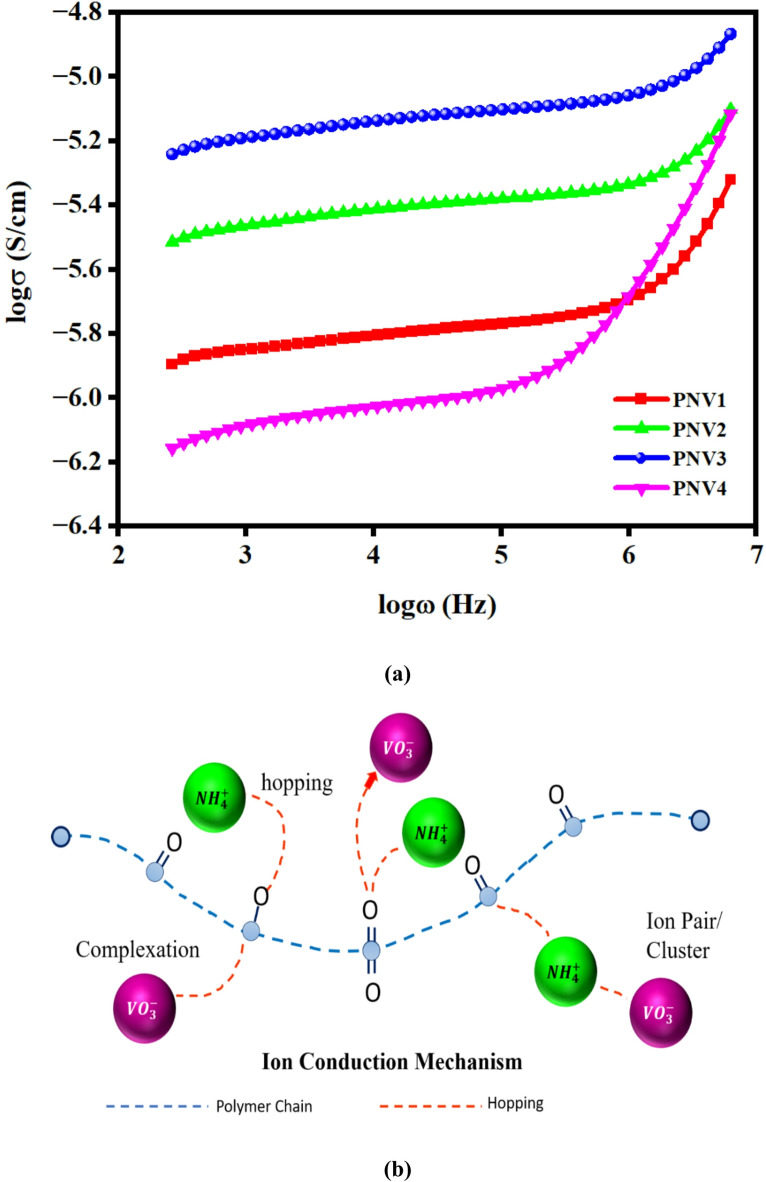
(**a**) Conductance spectra of PNV system at room temperature, (**b**) possible ion conduction mechanism.

The AC conductivity (σ_ac_) versus frequency response of PVA–sodium alginate polymer electrolytes doped with different concentrations of ammonium metavanadate (NH₄VO₃) is illustrated in Fig. 5(a). The plot of log(σ) vs. log(ω) reveal two distinct regions: a low-frequency plateau and a high-frequency dispersive slope. The plateau corresponds to DC conductivity (σ_dc_) resulting from long-range ion transport, while the rise at higher frequencies indicates localized ion hopping between neighbouring sites^47^.

As the salt concentration increases from 5 wt% to 15 wt%, there is a significant increase in σ_dc_, which is attributed to the growing number of mobile NH₄⁺ and VO₃⁻ ions. These ions interact with polar functional groups (–OH and –COO⁻) within the polymer matrix, facilitating transient ionic pathways and enhancing conductivity^48,49^. This behavior is consistent with the mechanism proposed by Jonscher, where ion mobility in disordered polymer systems increases with salt content up to an optimal concentration^50^.

However, at 20 wt% NH₄VO₃, the conductivity decreases, likely due to ion–ion aggregation and reduced polymer flexibility, which limit the number of free charge carriers. This trend has been widely reported in polymer electrolytes, where excessive salt leads to ion pairing or clustering, negatively impacting ion transport^51^. Additionally, the high-frequency tail seen in all spectra confirms electrode polarization behavior and hopping conduction, supporting the ion-dominated transport model^52^.

##### (b) Ion conduction mechanism

The conduction behavior observed in the NaAlg: PVA: NH₄VO₃ polymer electrolyte system shown in Fig. 5 (a) follows a hopping-based ion transport mechanism, where ions traverse between transient coordination sites supported by the segmental motion of the polymer chains. The incorporation of NH₄VO₃ acts not only as a source of mobile ions (NH₄⁺ and VO₃⁻), but also alters the structural organization of the polymer matrix by reducing crystallinity and promoting the formation of amorphous domains, thereby increasing the free volume and enhancing ionic conductivity^53^.

The overall ion conduction process shown in Fig. 5 (b) involves: (i) Complexation of NH₄⁺ ions with polar oxygen-containing groups such as –OH and –COO⁻ in the polymer backbone, (ii) Ion dissociation, yielding free NH₄⁺ and VO₃⁻ ions, which serve as charge carriers, (iii) Localized ion hopping between temporary coordination sites facilitated by polymer segmental motion^54^. At higher salt concentrations (e.g., 20 wt%), ion pairing or aggregation occurs, which limits the number of free charge carriers and hinders conductivity due to reduced ion mobility and polymer chain flexibility^55^. Thus, the optimal NH₄VO₃ concentration of 15 wt% achieves the right balance between ion availability and polymer chain dynamics, maximizing conductivity. This composition is particularly suitable for next-generation solid-state electrochemical energy storage devices, including batteries and supercapacitors, where safety, flexibility, and efficiency are critical^21^.

####  Dielectric spectra analysis

**Fig. 6:**
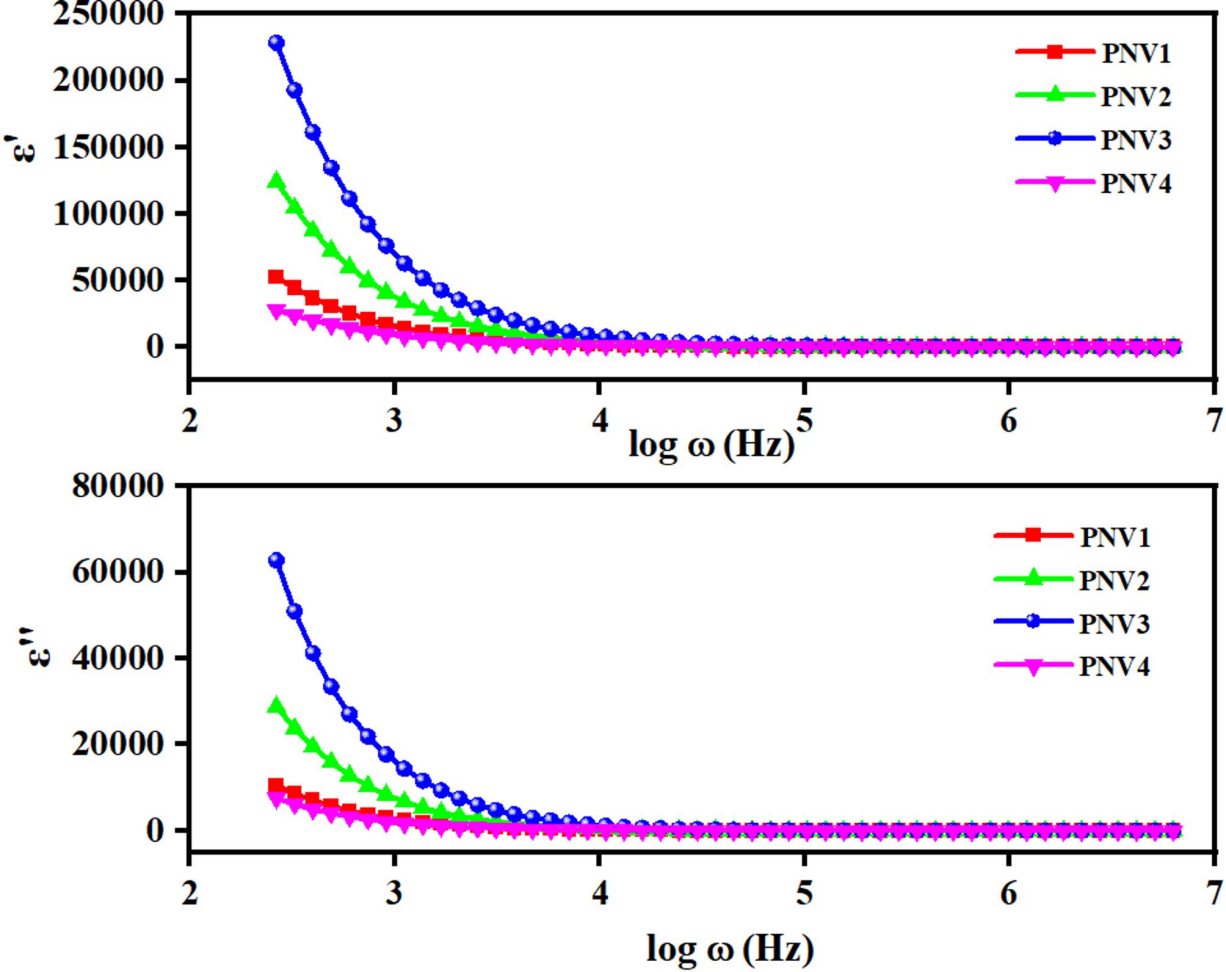
Dielectric constant $\:{\:\epsilon\:}^{{\prime\:}}\left(\omega\:\right)$versus log {ω(Hz)} and Dielectric loss $\:{\:\epsilon\:}^{{\prime\:}{\prime\:}}\left(\omega\:\right)$versus log {ω(Hz)} of PNV (PVA: NaAlg: NH_4_VO_3_ (5, 10, 15, 20 wt%) polymer blend electrolytes.

Figure 6 shows real (ε′) and imaginary (ε″) portion of the dielectric permittivity as a function of log ω for divulging the dielectric capabilities of NaAlg: PVA: NH₄VO₃ polymer blend electrolytes. The values of both ε′ and ε″ both are extremely large at low frequencies (log ω = 2.5–3), and the value decreases with increase in frequency. It arises due to interfacial polarization and space-charge effects, which are characteristic of the salt-doped polymer. In Fig. 6, the real (ε′) and imaginary (ε″) components of the dielectric permittivity as a function of log ω are displayed to illustrate the dielectric characteristics of NaAlg: PVA: NH₄VO₃ polymer blend electrolytes. Both ε′ and ε″ exhibit extremely high values at low frequencies (log ω = 2.5–3), which gradually decline with increasing frequency. Interfacial polarization and space-charge effects, which are common in salt-doped polymer electrolytes, are the cause of this pattern^56^.

The samples with the highest ε′ at low frequencies are those with a 15 weight% NH_4_VO₃ composition. This suggests that optimum dipolar orientation and free charge carrier density are ensured with the salt loading modesty that enhances dielectric responsiveness^57^. Figure 6 displays the dielectric properties of NaAlg: PVA: NH_4_VO₃ polymer blend electrolytes which shows the real (2) and imaginary (2) parts of the dielectric permittivity as a functional dependence on log ω. At frequencies as low as (log ω ≈ 2.5–3) very high values of both the ε′ and ε″ imply that at that frequency, the dielectric constant and the loss are incredibly high and decrease rapidly with increasing frequency. This trend can be attributed to interfacial polarization and space-charge effects which are characteristics of salt-doped polymeric electrolytes^56^.

Of all the samples, 15 wt% NH_4_VO₃ composition has maximized values of ε′ and ε″ at low frequencies (log ω ≈ 2–3) and is followed by 10 wt%, 5 wt%, and 20 wt%. This indicates that moderate salt loading optimizes magnitude of dipolar orientation and concentration of free charge carriers thereby enhancing dielectric performance^54^. At higher frequencies (log ω ≈ 5), ε′ and ε″ vary less and converge to smaller, constant levels of the dipole model and ion model failure to respond to high frequency fields, which has been reported widely in polymer blended electrolytes^56,58^.

The ε″ peak intensity is most pronounced for the 15 wt% sample, implying greater dielectric loss due to ion–dipole relaxation mechanisms. Meanwhile, the 10 wt% sample shows moderate dielectric loss, reflecting efficient ion mobility with minimal clustering, which may promote superior ionic conductivity^57^. Such behaviour is consistent with those obtained in PVA–K₂CO₃ gel polymer electrolytes where both ε′ and ε″ of the composite decreased with the frequency and high conductive composites were associated with a larger dielectric response at low frequencies^58^.

Therefore, the 15 wt% NH accent configurations provide the highest dielectric polarization whereas 10 wt% represents a concession which is reasonable in connection with the ion dissociation and the chain flexibility of the polymer necessary in connection with the energy storage devices.

**Fig. 7:**
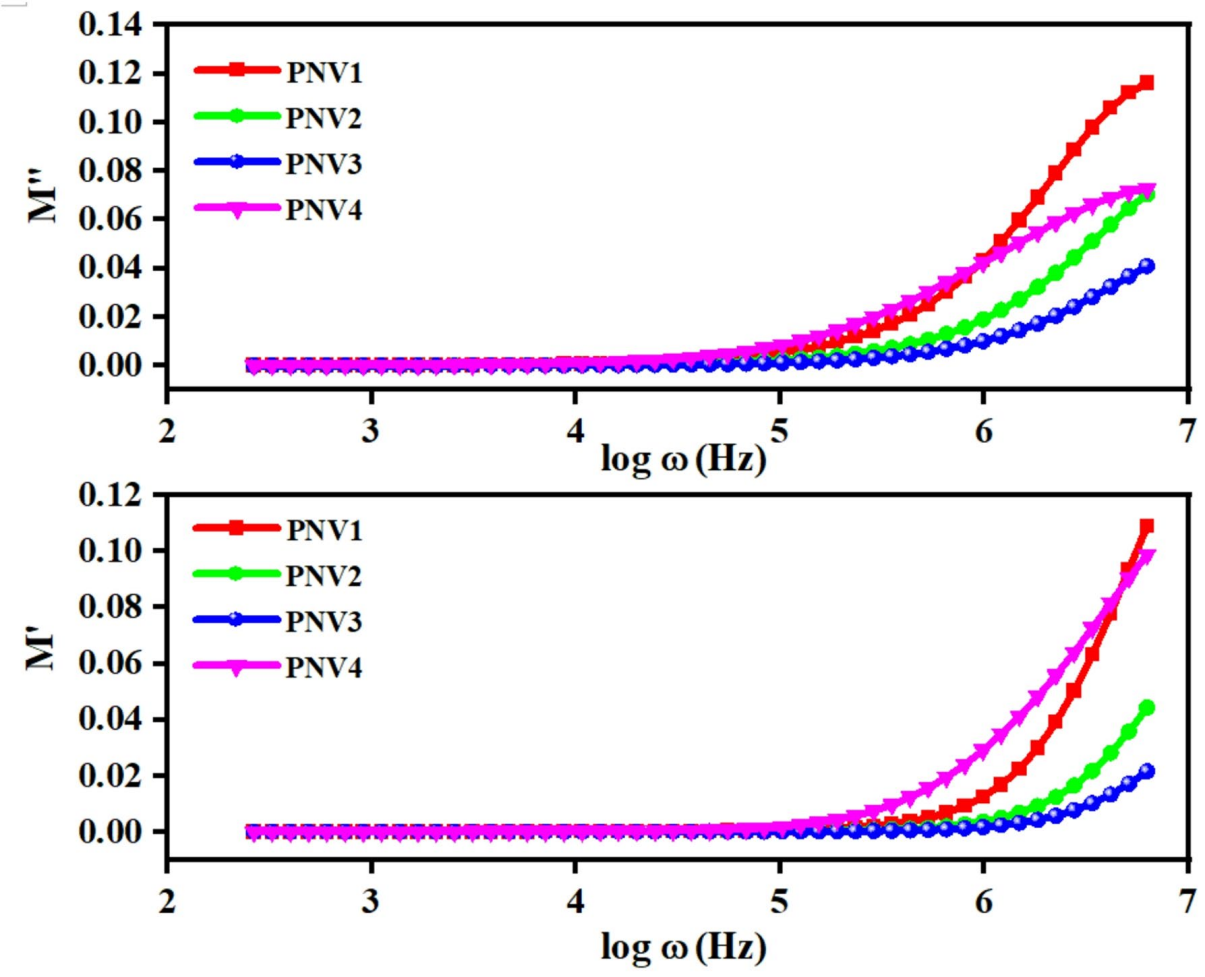
Real part of electric modulus $\:{\:M}^{{\prime\:}}$versus log ω and Variation of the imaginary part of electric modulus $\:{\:M}^{{\prime\:}{\prime\:}}$versus log ω of PNV (PVA: NaAlg: NH_4_VO_3_ (5, 10, 15, 20 wt%) polymer blend electrolytes.

Figure 7 shows real (M′ ) and imaginary (M″) components of electric modulus as a function of log of frequency in NaAlg: PVA: NH_4_VO_3_ (PNV) electrolytes with salt loading of 5, 10, 15 and 20 wt%. Both, M′ and M″ are approached by log ω < 5 with values close to zero at low frequencies which signal the relatively modest polarization of the electrodes and space charge behaviour of ionic conductive polymer matrices^59^. At higher frequencies, above log ω ≈ 5, M′ rises sharply drops off in a steep fashion and the dielectric stiffness begins to occur with considerable ionic mobility limitation. Particularly, in the 15 wt% NH_4_VO_3_ sample, M′ values remain the lowest and the M″ peak the slowest to emerge meaning that the polymer exhibits an increased segmental dynamics and overall shorter response time, a characteristic of better ionic conductivity^60^. Conversely, M′ at low frequencies is higher in the 5 wt% and 20 wt% samples, suggesting that the ion cluster or a slow chain mobility, a situation that impedes ionic motion. This attribute is an indication of the consequence of improperly salting the polymers either below or above on their flexibility and ion separation^61^. A combination of these inferences confirms that 15 wt% NH_4_VO_3_ is the best balance of ion dissociated and flexibility in the polymer. The results affirm better ion conduction parameters within the host matrix of NaAlg: PVA.

#### Tangent spectra analysis

**Fig. 8:**
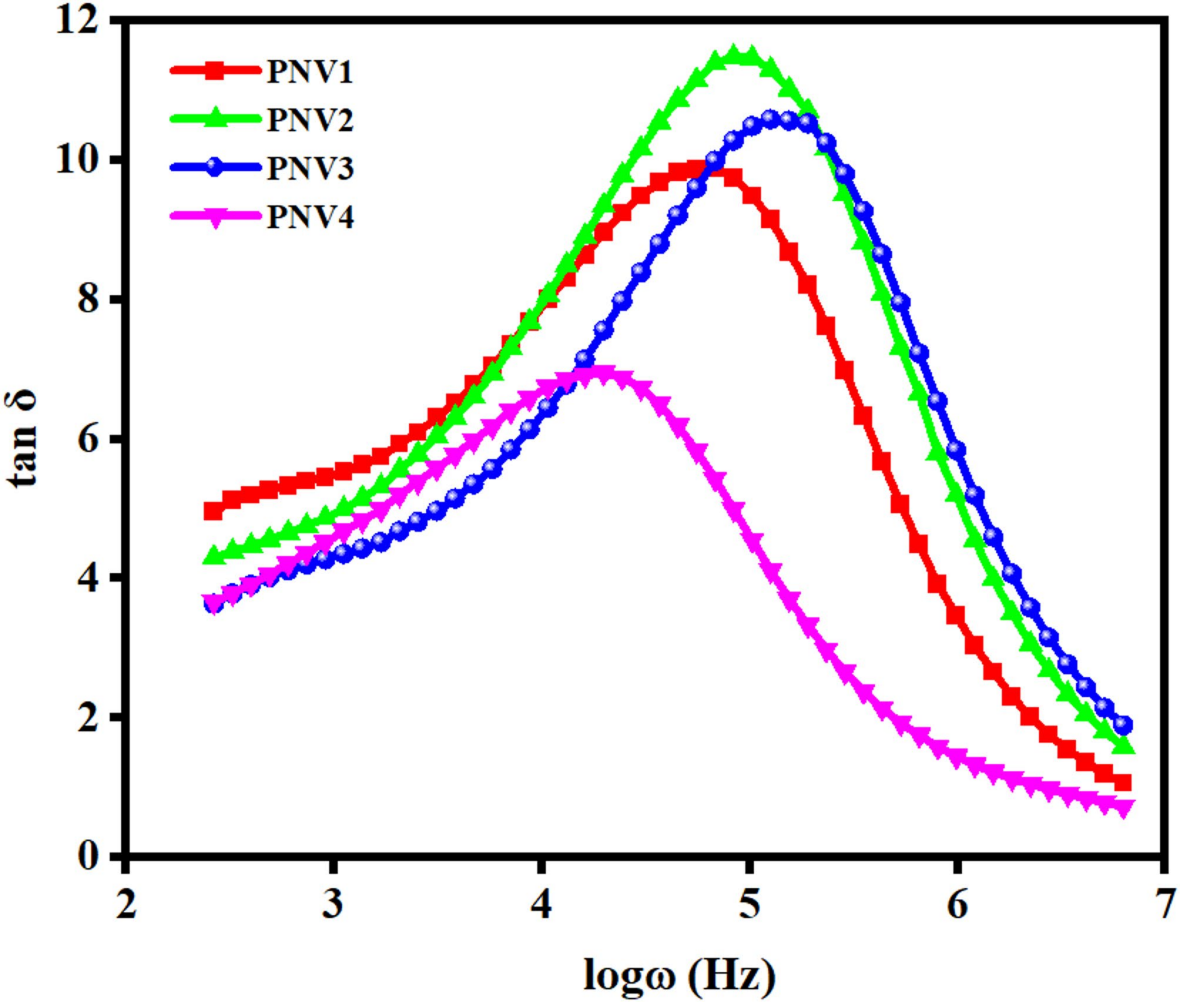
Loss tangent spectra for PNV blend electrolytes of different wt %.

Figure 8 is the plot of dielectric loss (tan δ) with respect to frequency (log ω) in polymer nanocomposite electrolytes doped with different concentrations of NH₄VO₃. All the samples show a single peak in the relaxation spectra, suggesting that the main mechanism of relaxation is the polymer segmental motion coupled with the transport of mobile ions. Among the samples with higher dielectric loss peaks, it is remarkable that 15 wt% NH₄VO₃ has the lowest relaxation time of 6.76 × 10^− 6^ s, which can be attributed to the optimum amorphous phase content and the maximum polymer chain flexibility. This is in agreement with earlier results where, if the salt concentration used to modify polymer electrolytes was moderate, it would result in higher dipole relaxation and ion dissociation and therefore, greater dielectric loss^62^.

The peak shift of tan δ to higher frequencies with increasing filler content above 15 wt% (i.e., 20 wt%) indicates an increase in ion mobility as ion pair aggregation or stiffening of the polymer takes place. On the other hand, the lower concentrations (5 and 10 wt%) do not have adequate mobile charge carriers to achieve optimal performance. Therefore, it could be concluded that 15 wt% NH₄VO₃ was found to be the best blend composition to produce the maximum value of dielectric relaxation and ionic conductivity. The peak-centered dielectric characteristics and optimal filler loadings were also observed in other PVA-based systems and NaAlg-based systems in applications to energy storage devices^63,64^.

### Cyclic voltammetry analysis

**Fig. 9:**
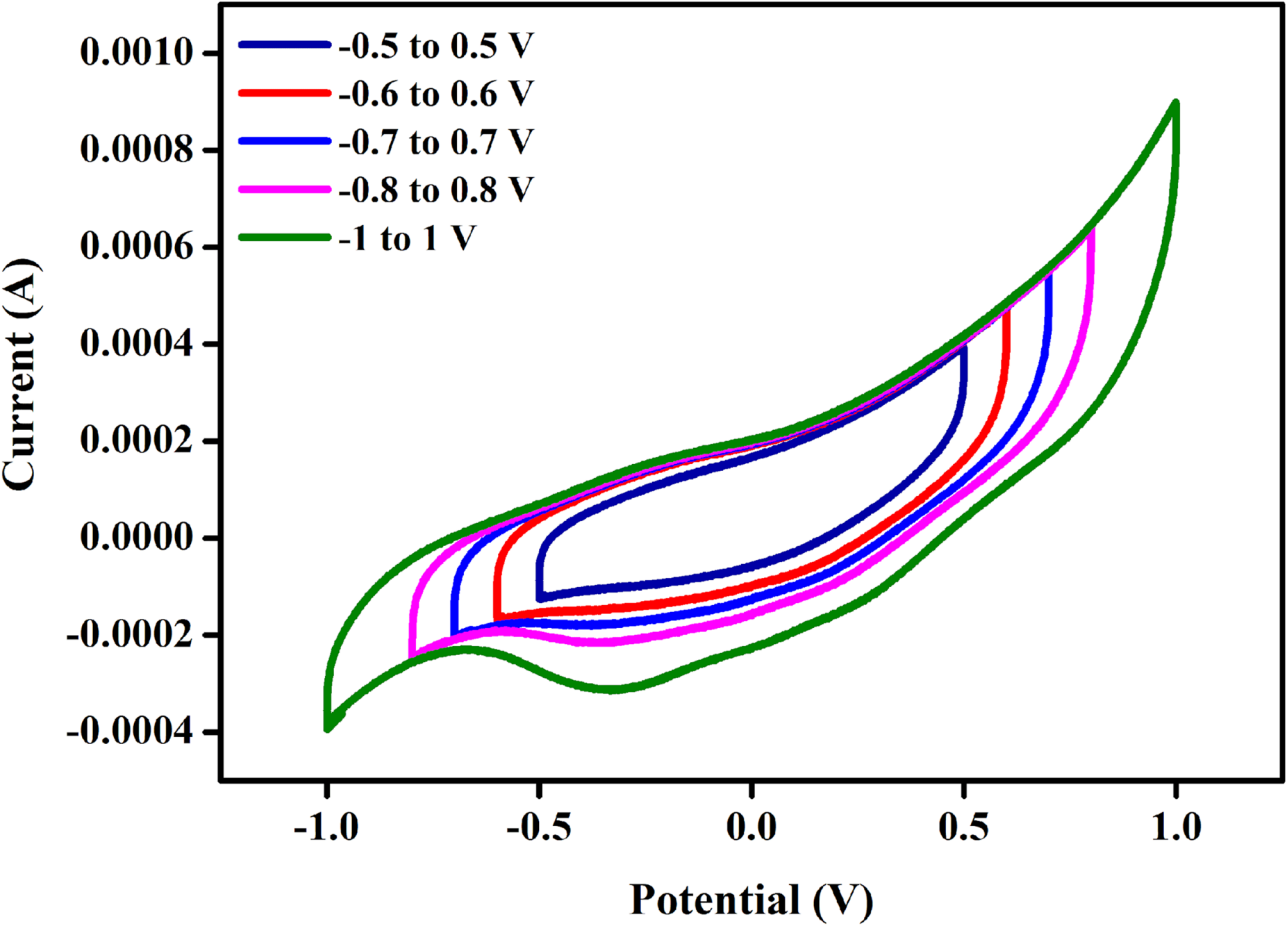
Cyclic Voltammetry for the higher ionic conductivity sample of PNV3 at different potential.

**Fig. 10:**
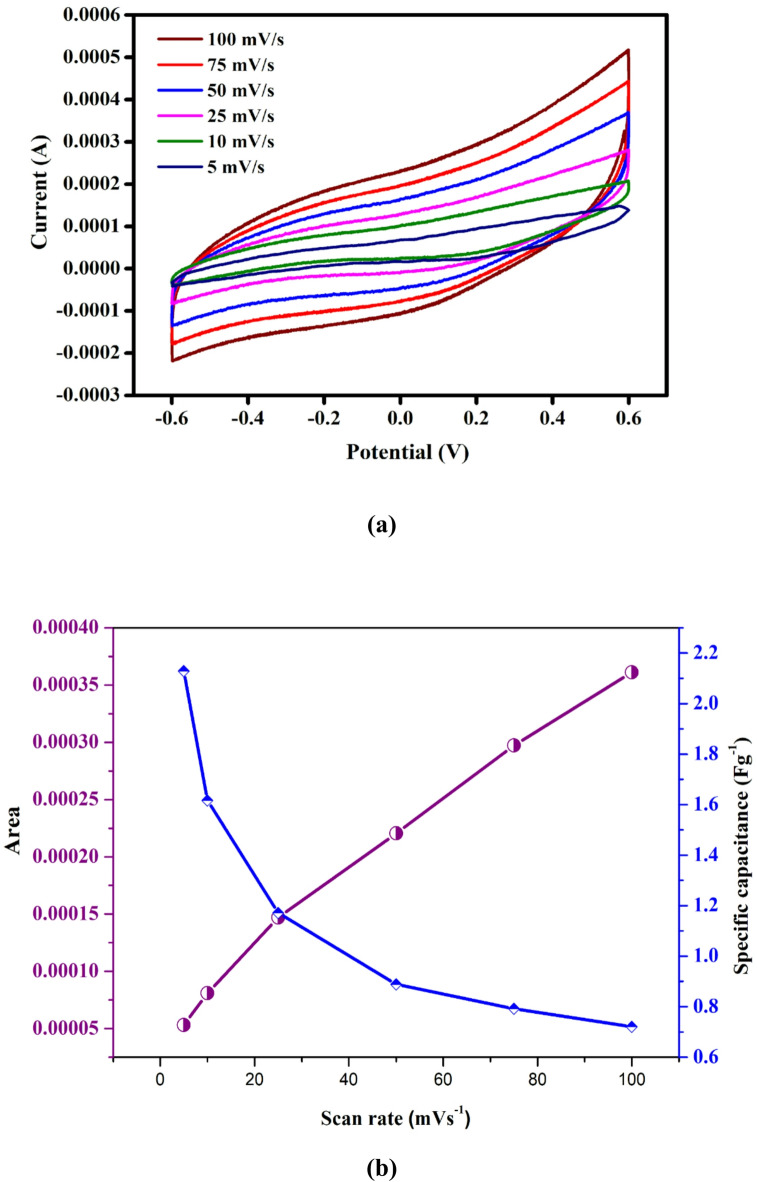
(**a**) Cyclic Voltammetry for the higher ionic conductivity sample of PNV3 at different potential. (**b**) Scan rate vs. area of PNV3.

Figure 9 shows the cyclic voltammetry curve of the PVA (60 wt%) : NaAlg (40 wt%) + 15 wt% NH₄VO₃ electrolyte. The CV curve exhibits a nearly rectangular shape, indicating good capacitive behavior and stable electrochemical performance of the cell. Voltammograms obtained at scan rates between 5 and 100 mV s⁻¹ shown in Fig. 10a displays curves that were generally close to rectangular, but with slight distortion, suggesting that the charge storage mechanism is not purely electric double-layer in nature and that additional Faradaic contributions are present^65,66^. The minor broadening observed in certain regions of the curves is consistent with redox activity linked to vanadium species from the ammonium vanadate component^67^.

Specific capacitance (Cs​) values with different scan rates (Table 6), calculated by integrating the CV curve area shown in Fig. 10b and normalizing to the active material mass, decreased systematically with increasing scan rate. The highest capacitance of 2.129 F g⁻¹ was recorded at 5 mV s⁻¹, whereas at 100 mV s⁻¹ the value dropped to 0.721 F g⁻¹. This decline indicates that ion transport and interfacial charge transfer processes are increasingly restricted at higher sweep rates, limiting the active area available for charge storage^68– 70^. At slower scan rates, electrolyte ions have sufficient time to diffuse through the polymer matrix and reach both surface and bulk active sites, thereby enhancing the total stored charge.

The observed behavior reflects a combination of electric double-layer charging from the PVA–NaAlg host matrix and pseudocapacitive contributions from ammonium vanadate. PVA’s semi-crystalline segments can restrict segmental chain motion, while NaAlg provides hydrophilic ionic pathways^71^. Ammonium vanadate offers redox-active V⁵⁺/V⁴⁺ centers, which can enhance capacitance at low scan rates but are subject to diffusion limitations^66,68^. This hybrid charge storage mechanism explains the higher capacitance at low rates and the pronounced drop at high rates, a pattern similar to other polymer electrolytes containing redox-active additives^72^.

The capacitance retention ratio, defined as Cs (100 mV s^− 1^)/Cs(5 mV s^− 1^), was approximately 34%, confirming that a substantial fraction of the total capacitance is inaccessible during fast cycling. Such rate-dependent behavior has been reported for polymer blend electrolytes with slow ion migration and significant interfacial resistance^66,70^. Additional studies, such as electrochemical impedance spectroscopy and the separation of capacitive and diffusion-controlled current contributions using power-law analysis, would provide further insight into the charge storage processes^73^.

**Table 6:** Specific capacitance of polymer electrolyte electrode at different scan rates.

Scan rate(mV/s)	Area	Specific capacitance (Fg^− 1^)
100	3.612 × 10^− 4^	0.721
75	2.973 × 10^− 4^	0.792
50	2.205 × 10^− 4^	0.889
25	1.469 × 10^− 4^	1.171
10	8.096 × 10^− 5^	1.617
5	5.321 × 10^− 5^	2.129

### Galvanostatic charge-discharge (GCD)

**Fig. 11:**
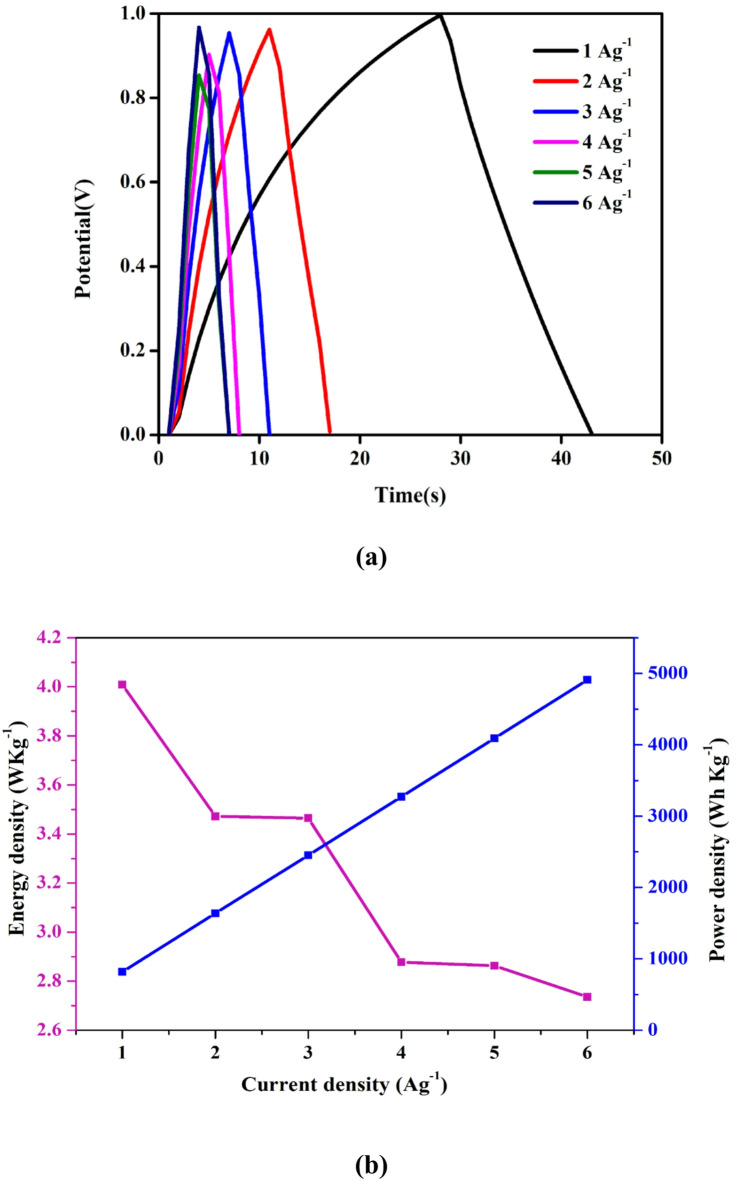
(**a**) GCD profile of EDLC at different potentials and (**b**) Energy density vs. power density of EDLC at different current densities.

Figure 11 (a) displays the galvanostatic charge–discharge (GCD) curves for the PNV3 polymer electrolyte-composed of PVA, sodium alginate, and 15 wt % NH₄VO₃ recorded at current densities ranging from 1 to 6 A·g⁻¹. The curves exhibit nearly symmetrical triangular shapes, indicating ideal electric double-layer capacitance with negligible faradaic contribution, consistent with similar behavior observed in Alginate–PVA–LiTFSI systems showing predominantly ionic transport (t_ion_ ≈ 0.98) and low resistance^74^. At 1 Ag⁻¹, the charge–discharge duration is significantly longer-reflecting higher specific capacitance and effective ion diffusion. As current density increases, discharge time decreases due to limited ion transport time and less accessibility to active sites-typical in polymer electrolyte systems. Notably, the GCD profiles show minimal initial voltage drop (IR drop) even at high currents, indicating low internal resistance and high ionic mobility-paralleling findings in NH₄I-doped Alginate–PVA blends where plasticization lowered T_g_ and enhanced conductivity^75^. As shown in Fig. 11(b), the energy density versus power density plot confirms that the device maintains consistent energy storage and power delivery across different current densities. The maintenance of triangular shapes across all current densities further demonstrates electrochemical stability and structural integrity, much like PVA-based gel electrolytes with ammonium salts and ionic liquids, which preserve EDLC behavior and capacitance retention under cycling^76,77^. Overall, the GCD analysis confirms that the optimized PNV3 composition offers high ionic conductivity, low internal resistance, and stable EDLC performance, making it a promising candidate for flexible energy storage devices.

###  Transference number analysis

The ion transference number (t_ion_) was determined by Wagner DC polarization using blocking electrodes (silver coated with graphite). The transference number measurement (TNM) involves the use of either non-blocking or blocking electrodes. In non-blocking electrodes, both ions and electrons contribute to conduction, while in blocking electrodes, only electrons can move through them. To identify whether electrons or ions are the primary charge carriers, one can compare the electron transport time (t_e_) and the ion transport time (t_ion_). For instance, in a polymer electrolyte film, the ion transport time (t_ion_) is greater than the electron transport time (t_e_), indicating that ions are the main charge carriers in the polymer electrolyte^78^. The silver electrode coated with graphite is utilized to obstruct the ions. The high initial current occurs due to the flow of both ions and electrons, while the final saturation current is solely due to electron conduction. The transference numbers for the prepared electrolytes are provided in Table 7^79^.

The transference number has been calculated using the given formula:

6 $$\:{t}_{ion}=\frac{{I}_{i}}{{I}_{f}}$$

7 $$\:{t}_{e}=1-\:{t}_{ion}\:$$

where t_ion_ is the transport number of ion, t_e_ is the transport number of electron, Ii is the initial current, and If is the final current.

**Fig. 12:**
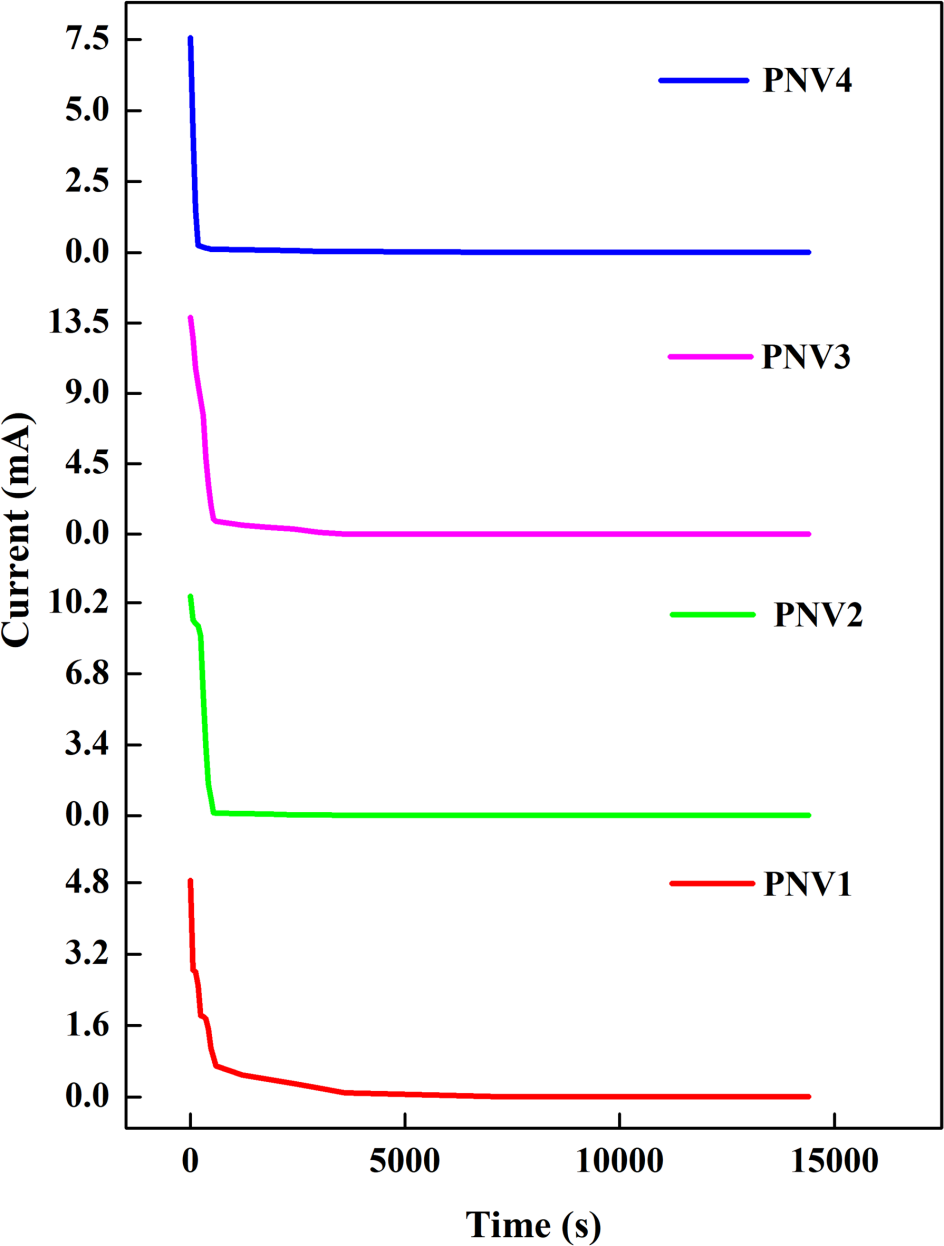
Wagner DC polarization curve of PNV1, PNV2, PNV3 and PNV4 at ambient temperature.

The current is observed as a function of time until a steady state is achieved. Initially, the current decreases over time, as illustrated in Fig. 12^80^.

The ion transport number rises with higher salt concentrations, which is attributed to the increase in the concentration of ions (both cations and anions), leading to a higher initial current. For the 15wt% NH_4_VO_3_ complexed electrolyte system, the ion transport number reached a high value of 0.99928, which may be adequate for the needs of solid-state electrochemical cells^81^ (Table 8).

**Table 7:** Calculated C_s_ values, energy density, and power density for PNV3 of the fabricated EDLC at different current densities.

Current density (Ag^− 1^)	Specific capacitances (Fg^− 1^)	Energy density (W kg^− 1^)	Power density (Wh kg^− 1^)
1	35.636	4.009	818.18
2	30.848	3.472	1636.36
3	30.808	3.465	2454.54
4	25.575	2.877	3272.72
5	25.454	2.863	4090.91
6	24.323	2.736	4909.09

**Table 8:** Transference number of the prepared electrolyte (PNV1, PNV2, PNV3 & PNV4).

S.No	Sample code	Weight% of salt	t_ion_	t_e_
1.	PNV1	5	0.997938	0.002062
2.	PNV2	10	0.999049	0.000951
3.	PNV3	15	0.999278	0.000722
4.	PNV4	20	0.998677	0.001323

### Linear sweep voltammetry (LSV)

**Fig. 13:**
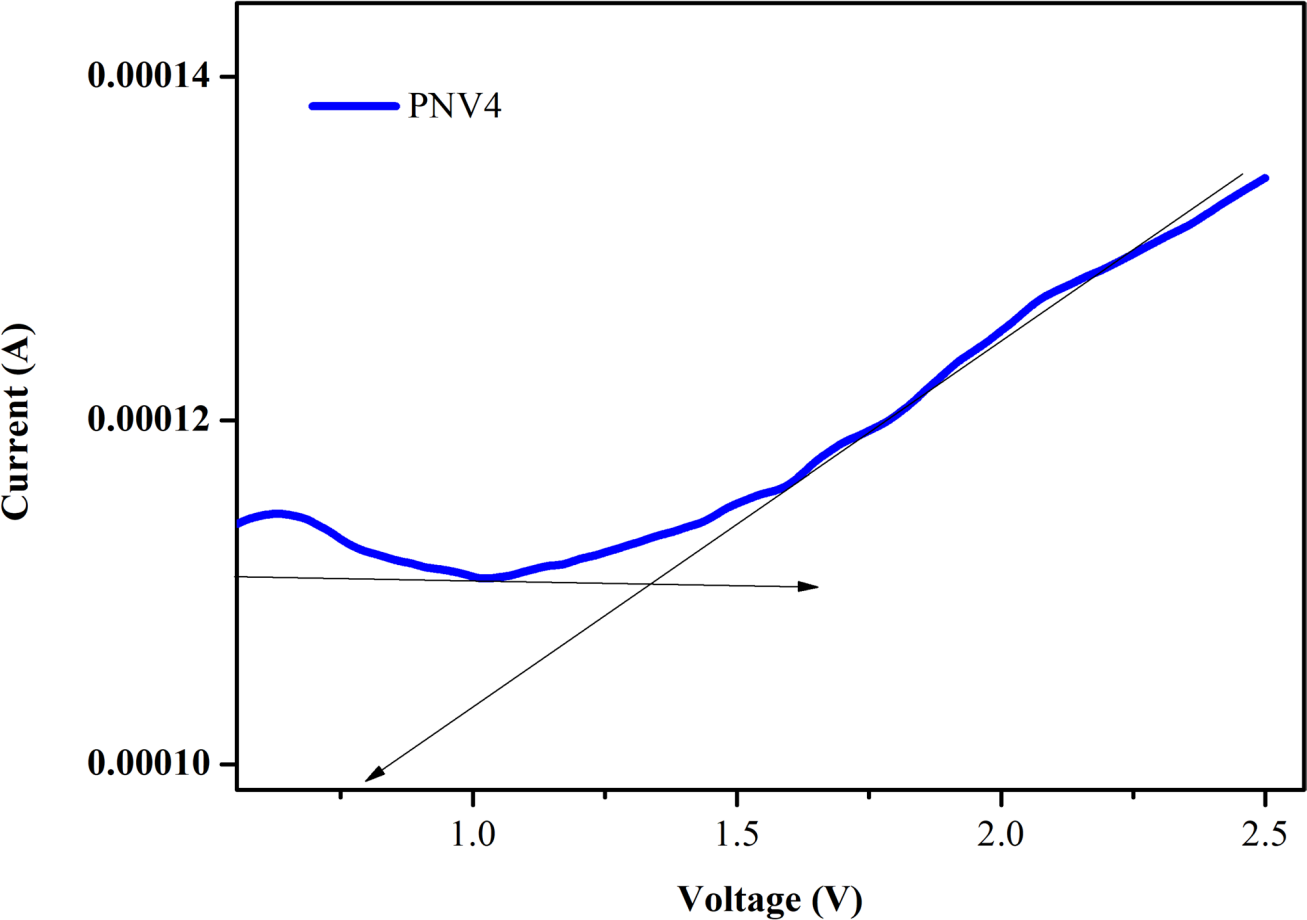
Linear sweep voltammetry for the higher ionic conductivity sample PVA: NaAlg:15 wt% NH_4_VO_3_.

LSV is used to assess the electrochemical stability potential window of polymer-blend solid electrolytes. The LSV analysis was conducted on the PVA: NaAlg/NH4Cl system, which exhibited the highest conductivity, to evaluate its electrochemical stability. For the PVA: NaAlg with 15 wt% NH_4_VO_3_, which is the most conductive solid polymer electrolyte (SBPE), LSV was utilized to determine the electrochemical stability window (ESW), as illustrated in Fig. 13^82^. The setup involved a silver electrode on both sides of the PVA: NaAlg:15 wt% NH_4_VO_3_ layer. The voltage was applied in the range of 0 to 1.2 V. No significant current was detected up to an electrode potential of 1 V. However, beyond the breakdown voltage of 1.34 V, a sharp increase in current was noted, indicating the presence of the electrochemical stability window. This increase was attributed to the decomposition of the SBPEs at the interface with the inert electrode^27,83^.

## Conclusions

In this study, NaAlg–PVA polymer electrolyte films doped with ammonium metavanadate were successfully fabricated via the solution casting method. The systematic increase in ionic conductivity with NH₄VO₃ addition up to 15 wt% highlights the effective role of salt in modulating charge carrier dynamics. The decrease in crystallinity seen in XRD patterns, along with FTIR-confirmed polymer–salt interactions, supports the improved amorphous nature of the film — a key factor in enhancing conductivity results of Ionic conductivity, relaxation time, and dielectric studies, CV have been reported. Interestingly, the specific capacitance exhibited a non-linear trend with current density in GCD analysis, initially decreasing and then increasing, which could be attributed to the interplay between charge storage efficiency and ion accessibility at different current regimes. Impedance analysis has confirmed that the NaAlg/PVA system with 15 wt% of NH_4_VO_3_ exhibits the highest ionic conductivity, measuring 0.67 × 10^− 5^ S cm^− 1^. Additionally, this optimized blend containing 15 wt% of NH_4_VO_3_ resulted in a shift of the peak in the loss tangent plot to higher frequencies, accompanied by a reduced relaxation time of 6.76 × 10^− 6^ s. The electrochemical properties were further validated through cyclic voltammetry (CV), and the LSV investigation of NP4 showed a breakdown voltage of 1.34 V. This behaviour suggests an adaptive charge-discharge mechanism that may be beneficial in variable-load conditions. Overall, the electrolyte system demonstrates a viable path forward for flexible energy devices, combining mechanical integrity with improved electrochemical performance. Future work may focus on 0optimizing salt concentration and exploring alternative plasticizers or nanofillers to further boost performance for real-world applications.

## Data Availability

The datasets used and/or analysed during the current study available from the corresponding author on reasonable request.
